# “It Made a Difference to Me”: A Comparative Case Study of Community Pharmacists’ Care Planning Services in Primary Health Care

**DOI:** 10.3390/pharmacy7030090

**Published:** 2019-07-11

**Authors:** Theresa J. Schindel, Rene R. Breault, Christine A. Hughes

**Affiliations:** Faculty of Pharmacy and Pharmaceutical Sciences, University of Alberta, 3-171 Edmonton Clinic Health Academy, 11405 87 Avenue NW, Edmonton, AB T6G 1C9, Canada

**Keywords:** pharmacist services, community pharmacy, care plan, compensation, primary health care, information sharing, qualitative research, comparative case study, value

## Abstract

In some jurisdictions, governments and the public look to community pharmacies to provide expanded primary health care services, including care plans with follow-up. Care planning services, covered by the Compensation Plan in Alberta, Canada, require pharmacists to assess an eligible patient’s health history, medication history, and drug-related problems to establish goals of treatment, interventions, and monitoring plan. Follow-up assessments are also covered by the Compensation Plan. A comparative case study method facilitated an in-depth investigation of care planning services provided by four community pharmacy sites. Data from 77 interviews, 61 site-specific documents, and 94 h of observation collected over 20 months were analyzed using an iterative constant comparative approach. Using a sociomaterial theoretical framework, the perceived value of care planning services was examined through an investigation of the relationships and interactions between people and information. Patients perceived the value of care planning as related to waiting time to access care and co-creating individualized plans. Physicians and other health care professionals valued collaboration, information sharing, and different perspectives on patient care. Pharmacists valued collaboration with patients and other health care professionals, which renewed their sense of responsibility, increased satisfaction, and gave meaning to their role.

## 1. Introduction

Primary health care is based on the principles of equity, collaboration, and community participation [1]. It has been defined as the first place individuals go to receive health care services [2]. Attributes of primary health care include patient and family centeredness, continuity of relationships, and information continuity and management [3]. The delivery of primary health care involves teams of health care providers; the exact composition of these teams depends on patients’ needs. As team members, pharmacists do their part to meet patients’ needs and provide essential drugs. In many parts of the world, pharmacists are becoming increasingly involved in the delivery of primary health care, contributing to chronic disease management and providing immunization services [4,5] and the community pharmacy is a place that the public would go to receive some primary health care services [6,7]. Describing community pharmacists as “primary care pharmacists” recognizes not only their accessibility to patients, but also their contributions as team members delivering primary health care services including management of chronic conditions such as hypertension, treatment of minor ailments, administration of vaccinations, and triaging and referring patients with acute conditions to emergency departments or family physicians [8,9]. Governments, such as those in Canada and Alberta, and the public therefore look to community pharmacists as providers of primary health care services [2,10].

Research examining pharmacists’ preferences for existing and potential roles indicates a preference to provide more patient-centered services [11] and to contribute more to meeting primary health care needs in their communities through teamwork [12]. Pharmacists are now involved in providing enhanced services that support primary health care such as comprehensive medication reviews, immunizations, adherence packaging, and development of comprehensive care plans with follow-up [13].

A care plan is an essential component of the patient care process [14] and refers to a detailed document prepared by a pharmacist that outlines the pharmacist’s and patient’s responsibilities to resolve drug therapy problems or health needs to achieve the patient’s health goals, and to prevent potential drug therapy problems. Care plans are developed with input and participation of the patient and can be developed collaboratively with other health care providers involved in the patient’s care [15]. Documented care plans are fundamental to delivery of pharmaceutical care services [14,16], quality care, and communication with team members [17]. For example, care plans for chronic disease management can improve information sharing, enhance patients’ engagement in self-management, and promote collaboration among health care professionals [18]. Involvement in care planning services has the potential to increase the engagement of pharmacists in primary health care [19] and increase recognition of the value of pharmacists’ contributions to patient care.

Demonstrating value in primary health care addresses the triple aim of health systems to improve patient health outcomes, improve patient experience of care, and reduce health care costs [20,21]. Other approaches to show value acknowledge the role of care planning, along with access to care, continuity of care, relationships, evidence-based therapy, and patient engagement in the process [22]. Parameters that may be useful to demonstrate the value of care planning include patients’ experienced health, increased accessibility, building of trust, improved communication with care providers, relationship continuity of patients and care providers over time, information management [23,24], and experiences of health care providers including conditions that support health care providers to find meaning in their work [25].

Although care plans communicate value from perspectives of the effectiveness and cost-effectiveness of pharmacy services [26] and provide documentation that supports bi-directional communication of health care providers [27], very little research has been conducted regarding the perceived value of pharmacists’ care planning services. This study addresses this gap from the perspectives of patients and health care providers who have experience with care planning services. The overall objectives of this study are to explore how care pharmacists’ planning services covered by the Compensation Plan in Alberta were implemented and the perceived value of the care planning services. This paper aims to contribute to what is known about the perceived value of care planning services provided by pharmacists through community pharmacies.

## 2. Materials and Methods

### 2.1. Context

The setting for this research is the western-Canadian province of Alberta. Since 2006, pharmacists can access patient information including laboratory tests and records of dispensed medications through a provincial electronic health record known as Netcare [28]. The scope of practice for Albertan pharmacists enables them to order laboratory tests, administer drugs by injection, and prescribe drugs. Prescribing authority involves 3 types of prescribing: emergency prescribing, adapting prescriptions, and independent prescribing [29]. All pharmacists can prescribe in an emergency when patients cannot access other health services and all have authority to adapt a prescription initiated by another prescriber. When pharmacists prescribe by adapting a prescription, they may modify a prescription to extend therapy, change a dose or formulation. Pharmacists who have successfully applied for Additional Prescribing Authorization (APA) may also prescribe to initiate new drug therapy [29]. The APA application process involves peer review of a prescribing portfolio that includes narrative description of the pharmacists’ practice, documentation of patient care cases, and self-assessment of prescribing competencies [30]. Documentation of prescribing decisions is required along with communication with any other health professional whose care of a patient may be affected by the pharmacist’s prescribing decision [31]. At the time of this study, Alberta had a population of 4.2 million [32]. There were 5363 practicing pharmacists including 1658 with APA, 1377 registered pharmacy technicians, and 1232 licensed pharmacies [33]. Community pharmacy practice in Alberta varies from pharmacist to pharmacist within the scope of practice and their roles are continually evolving [34,35].

The Compensation Plan for Pharmacy Services, approved by the Government of Alberta and implemented in 2012, supports the scope of practice for pharmacists in Alberta by offering one of the most comprehensive fee schedules available for remuneration of pharmacy services in Canada [36]. It allows for payment to community pharmacies for a range of pharmacy services provided by pharmacists such as prescription renewals, administration of drugs and vaccines by injection, and care planning services, including follow-up assessments [37]. Since implementation, the number of compensated services increased from 30,000 per month (July 2012) to 170,000 per month (March 2016) and Comprehensive Annual Care Plans (CACP) and CACP follow-up assessments were among the top 8 services claimed by community pharmacies [36]. Similar to medication therapy management (MTM) services provided by pharmacists under the Medicare Modernization Act (2003) in the United States (US) [38], Albertan pharmacists involved in care planning services must assess a patient’s health and medication history, establish goals of treatment, suggest interventions, and implement a monitoring plan. To be eligible for a CACP covered under the Compensation Plan, patients must meet certain criteria; for example, they must have specific chronic diseases (e.g., diabetes) or have certain risk factors (e.g., tobacco use). 

CACP is defined as “a plan that is prepared and documented by a Clinical Pharmacist that documents the required elements” (see Table 1) [39]. The components of the plan are consistent with the patient care processes outlined by the National Association of Pharmacy Regulatory Authorities in Canada [15] and National Association of Joint Commission of Pharmacy Practitioners in the US [14]. Different from MTM services in the US, the CACP service criteria are fixed and the Alberta government pays pharmacies for the service as long as patients are covered by the provincial health care system (i.e., have an Alberta health care number). CACPs are developed by pharmacists in collaboration with the patient, after which all interactions are documented and shared with other health care professionals involved in the patient’s care [36]. Community pharmacies and pharmacists developed customized CACP templates as there are no prescribed care plan documentation templates. Patients must sign that they have reviewed and discussed their CACP with the pharmacist who prepared it as well as received a summary of the CACP [39]. Pharmacists with appropriate authorizations, including APA or injections, may implement interventions such as adjusting doses of chronic medications, prescribing smoking cessation therapy, or administering vaccines. Pharmacists may choose to suggest or discuss interventions with the patient’s physician or refer to other health care providers.

### 2.2. Theoretical Framework

**Figure 1:**
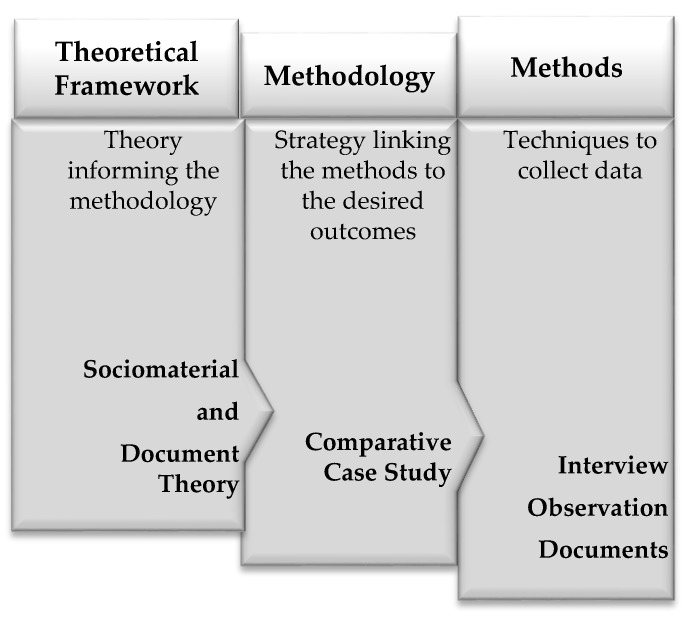
Elements of research used in this study.

The theoretical framework for this study draws on sociomaterial theory [40] and document theory [41], allowing us to explore the value of care planning services (Figure 1). A sociomaterial approach recognizes the importance of the social (i.e., relational) and material (i.e., care plans) aspects of everyday activities and their influence on our understanding of reality. A care plan, as a physical (material) document, combined with human (social) endeavors, serves to include, exclude and regulate actions [42]. Document theory considers a care plan as a source of information existing in 3 dimensions: material, social, and mental [41]. Thus, research based on document theory explores how these dimensions interact in different environments in which documentation takes place; in this study, document theory is applied in the context of care planning services associated with primary health care. In pharmacy practice, care plan documents themselves may be in the background when contemplating primary health care services. By acknowledging the role of these documents, this study brings care plans to the foreground of scholarly thinking, making them visible and asserting their value as part of the care planning services provided by pharmacists.

### 2.3. Methodology—Comparative Case Study

A comparative case study approach was used in order to gain an in-depth understanding of compensated care planning services within the real-world context of patient care in community pharmacy practice in Alberta [43,44]. The unit of study, or the case, was the CACP [45]. Case study research is ideal for exploring “how” and “why” research questions [45]. As part of larger study, the case study method was chosen to explore how pharmacists implemented care planning services and why services are perceived as valuable. Case study research may involve a single case or multiple case design [43]. This comparative case study was designed to include 4 different community pharmacy sites, or cases, a priori. The more sites included in a comparative case study, the greater the range of variation, thus the greater the contribution to what is known about care planning services in different contexts [43]. 

### 2.4. Methods

**Figure 2:**
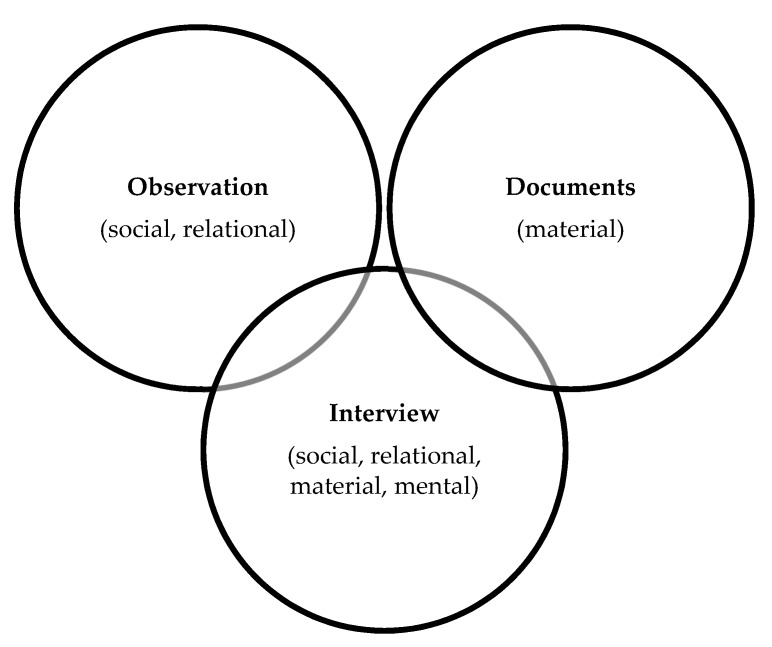
Methods of data collection used in this study. Interviews incorporated data from observation and documents.

Three methods of data collection were used in this study: interviews, observation, and documents. Interviews were semi-structured, consisted of open-ended questions, were conducted by members of the research team, were approximately 30–60 min in length, and were conversational in style (Appendix A, Table A1). One research team member was assigned to each site. The lead team member coordinated the site visit with the research assistant and both were involved in data collection. The research assistants were trained by the co-principal investigators. Most interviews were conducted in person at the sites. Some interviews with patients and physicians were conducted over the telephone following site visits. Interviews were audio recorded and transcribed verbatim. The second method of data collection, direct observation of the provision of patient care services, permitted the study of care planning within its natural setting or real-world context [45]. Researchers observed individuals involved in care planning services (pharmacists, pharmacy technicians, pharmacy staff, other health care providers, and patients), took field notes, and wrote a narrative description based on what was seen, heard, or sensed on site [44]. Observational data were collected at the time of the site visits using a pre-prepared form to guide documentation of observations and support reflexive research practices (Appendix A, Table A2). The research team developed the observation form to address the research questions. Observations focused on care planning interactions between pharmacists and patients, pharmacists and pharmacy staff, as well as development, storing and sharing of the care plan document. The researcher documented observations in a notebook, using the observation form as a guide. After the interaction concluded, the researcher transcribed notes to an electronic version of the observation form. A summary of interview questions related to observations were noted. Site-specific documents related to CACP services such as care plan templates and communication templates were also collected at site visits (Appendix A, Table A3). Data collected from observation and documents were incorporated into the interview process to invite materiality into interviews (Figure 2) [46]. 

### 2.5. Recruitment

The researchers identified types of community pharmacy sites where services covered by the Compensation Plan for Pharmacy Services were provided. Sites were selected by the research team using a purposive sampling technique [47] based on pre-defined variables [44]. The study included 4 community pharmacy sites where CACP services were provided, selected based on the following information: type of pharmacy (independent, franchise, corporate), population size (population centre) [48], volume of CACP services (number of reported CACPs provided per month), and provision of other compensated services (pharmacists with APA and injections authorization). A list of pharmacies and prescribing pharmacists provided by the Alberta College of Pharmacy and guidance from the Research Advisory Committee members were used to identify community pharmacy sites that represented pre-defined variables and likely to be engaged in care planning services under the Compensation Plan for Pharmacy Services. 

Pharmacy managers were initially approached by a member of the research team to discuss their pharmacy as a potential site for the case study. A total of 5 sites were recruited. One site withdrew from the study prior to the start of data collection due to unexpected staffing changes. All 4 cases involved community pharmacies and extended beyond the physical space to include patients, physicians, and other health care professionals involved in care planning services. Participants were recruited on site (patients, pharmacists, pharmacy technicians, pharmacy students, and staff) and in the local community (other health care providers involved with the pharmacist in the care planning process) [49]. Other health care providers included nurses, nurse practitioners, and physicians. Information posters about the study were displayed at the community pharmacies to indicate that researchers were on site and invited patients to approach researchers if they were interested in the study. Physicians associated with the community pharmacies and involved with care planning services were identified by the community pharmacists or patients and subsequently invited to participate in the study by the researchers. All participants provided informed consent. This research study was approved by the University of Alberta Health Research Ethics Board (Pro00059814).

### 2.6. Data Analysis

Data from interviews, observations, and documents collected at the sites were analyzed using an inductive constant comparative approach based on constructivist grounded theory [47]. Following transcription of the interviews, a member of the research team reviewed each transcript for accuracy and removed identifying information such as names of individuals, pharmacies, and towns or cities. The initial analysis entailed line-by-line coding of each phrase or portion of data from the various data sources by 1 research team member. All team members reviewed codes, emerging concepts, and supporting data following each site visit and at monthly research meetings to reach consensus. In keeping with the grounded theory approach, interview questions were adjusted to ensure an in-depth understanding of concepts (see Appendix A, Table A1). Separate analysis of data from each site permitted comparison of the data in terms of perceived value of care planning services. NVivo 12 software was used for storing data, coding, and analysis.

### 2.7. Rigour

The case study method is a rigorous qualitative research approach that ensures the trustworthiness of the results of research [50]. Elements of trustworthiness include credibility, dependability, confirmability, and transferability; these are parallel concepts to internal validity, reliability–reproducibility, objectivity, and generalizability, all concepts associated with the quality of quantitative research [51]. Trustworthiness was achieved through use of a combination of data sources (credibility), in-depth presentation of data and analysis (dependability), detailed documentation at all research stages, and sharing of research results with participants (confirmability). Based on this description of the study setting, readers can judge for themselves about the transferability of its results to other contexts.

### 2.8. Reflexivity

The roles and views of research team members were discussed throughout the research process: personal views and positions on the Compensation Plan for Pharmacy Services, the effect of care planning services on the pharmacy sites included in this study, the influence of archival documents and the media reports of compensated pharmacy services by the public and other audiences [52]. The research team addressed these issues reflexively at monthly research meetings held throughout the planning, data collection, analysis, and reporting phases of the project. In addition, reflexivity was incorporated in the form used to document observational data (Appendix A, Table A2). All members of the research team are associated with the pharmacy profession, 2 as practicing pharmacists (C.A.H., R.R.B.), but not involved in provision of compensated CACP services in community pharmacy practice.

## 3. Results

### 3.1. Sites

The four sites recruited for this study represented different practice contexts associated with CACP services. These included two independent pharmacies, a franchise pharmacy, and a large corporate chain pharmacy, all from population centers ranging widely in size [48,53]. Site 1 served a small population center as well as rural communities within 100 km of the community pharmacy. Site 2 served patients in an inner-city neighborhood in a large population center. Site 3 was located in a suburban area and routinely involved pharmacy students from the University of Alberta in care planning services. Site 4 provided CACPs for patients in care facilities outside of the pharmacy and worked closely with family physicians in primary care practices. At all sites, pharmacists integrated CACP services within their established practices. Sites 1, 2, and 3 developed approximately 20 CACPs each month, whereas Site 4 developed over 100 CACPs per month. Details of the sites are summarized in Table 2.

### 3.2. Data Collected

Data were collected over a period of 20 months between May 2016 and January 2018. Each site was visited three times over the course of a year, typically at 6 and 12 months following the initial visit. The data set included 77 interviews, 94 h of observation, and 61 site-specific documents. Table 3 summarizes the numbers of interviews and observation hours. Researchers collected document templates developed by each site to facilitate information gathering (e.g., care plan, patient assessment, medication history) and sharing (e.g., letters to physicians, medication lists, patient action plans). Types of documents collected are listed in Appendix A, Table A3.

### 3.3. Value of Care Planning Services

The value of care planning services was evident in the interactions between people and information. Six areas of value were identified: reinforcing patient-centered care, reducing waiting time for care, co-creating individualized plans with patients, collaborating with physicians and other health care providers, revealing possibilities for pharmacists’ contributions to primary health care, and making sense of pharmacists’ roles in primary health care. Results are presented in the sections below. They reflect a sociomaterial exploration of value, which emphasizes the importance of the social and material aspects of care planning services and how they are related to action in practice. Representative quotes from interview data are provided in Appendix A, Table A4.

#### 3.3.1. Reinforcing Patient-Centered Care

*“I like the structure and deliberateness… [it] is a remarkable way to connect with people and to help them manage their health. It’s been really good that way to make that a deliberate process”*

*(Site 2, Pharmacy Manager)*

The care plan document focused pharmacists’ and pharmacy technicians’ work to support the patient care processes. Value was associated with the changes made to work arrangements in everyday practice so that pharmacists could “sit down in the room”, “connect with patients”, and complete the “whole care plan process”. To accomplish this, pharmacists physically excused themselves from dispensing and other duties to prioritize care plan development and follow-up. Other pharmacists and pharmacy technicians or assistants at the sites adjusted their work to accommodate. At Site 4, pharmacists were specifically assigned to provide care planning services exclusively. Modifications to the duties of the pharmacy technician at Site 1 included identification of patients who presented to the pharmacy with questions about their care plans or patients who were eligible for care planning services based on the Compensation Plan (i.e., the CACP).

Pharmacists used care plan templates to structure care planning services. These templates guided the processes of gathering information, identifying drug-related problems, obtaining information to support drug-therapy decisions, preparing documents for physicians and patients, scheduling follow-up interviews or monitoring, and updating patient information systems. Information gathering from the patient’s electronic health records and pharmacy system was usually completed prior to the care plan meeting with patients and documented on the CACP template. The time required for the CACP varied from pharmacist to pharmacist, sometimes taking up to 4 h to complete the process. Care plan documents were valued for their ability to guide changes in work and reinforce the focus of the work on patient care services

#### 3.3.2. Reducing Waiting Time for Care

*“I waited actually over 15 months to see a specialist… The pharmacist in five minutes told me more than that specialist did, and there was no waiting period.”*

*(Site 3, Patient 1)*

Patients valued care planning services, which saved time and increased convenience. Patients associated the CACP with timely access to care. Access to care at the pharmacy was more convenient than scheduling appointments at physicians’ offices. Patients compared their prior experiences in both environments, noting lengthy waiting times to see their physicians, less comfortable medical office environments, and greater formality than at the pharmacy. Access to care was not only easier; the community pharmacy environment was more conducive to conversations about health and concerns. Patients noted that their CACP interviews with pharmacists were less formal than physician interviews. Speaking to their pharmacists was like speaking to “family”. Reduced waiting time to see a pharmacist led patients to seek advice and answers to questions previously directed to their physicians. For example, they might ask a pharmacist to determine if a visit to a physician or hospital emergency room was required. They also sought information about their health conditions, looking to pharmacists for education about various medical issues. The value of the care plan for patients was thus associated with reduced waiting time and convenient access to care.

#### 3.3.3. Co-Creating Individualized Plans with Patients

*“It made a difference to me. I mean, I appreciate it. I appreciate the chance to go over my meds and things that bother me at the time or what we could do about one thing or another.”*

*(Site 1, Patient 4)*

The initial development of the CACP promoted relationship building between patients and pharmacists. Subsequent patient–pharmacist interactions arising from CACP monitoring plans and scheduled follow-up reinforced previously established relationships and improved rapport. Some patients initiated follow-up with pharmacists as new information or questions arose. Through frequent and continuous conversations, individualized plans were created with patients’ willing participation in the process. These interactions frequently involved the patient and pharmacist both interacting with others, such as the patient’s family members or caregivers, physicians, or nurse practitioners. In developing CACPs, pharmacists and patients also interacted with information and other material objects such as weigh scales, blood pressure monitors, insulin pumps, and electronic resources. Following the CACP interview, patients were provided with a current list of medications. Patients who left with an understanding of their goals and action items associated with the CACP were motivated to engage further in their care. However, not all patients left the CACP experience with a CACP document and a clear understanding of their goals and action plan. Through involvement in the care planning, patients’ engagement with their care was enhanced. Patients valued the time spent with pharmacists, noting that pharmacists took “time to listen”, and valued knowing more about their care and a sense of their responsibilities for action.

The CACP was more than a document that recorded important information about patients and their goals. It caused things to happen and made a difference in patients’ experiences with health care services. Patients valued care planning services for emotional reasons as well as practical ones. Patients valued the CACP for the “peace of mind” and “secure feeling” they got after using the services. Value was therefore derived from patients and pharmacists working together to create care plans and patients’ experiences of care provided by pharmacists.

#### 3.3.4. Collaborating with Physicians and Other Health Care Providers

*”It’s a wonderful adjunct to my practice. It makes my practice better… It makes me think about things in a different way.”*

*(Site 4, Physician 2)*

Information sharing is essential to the collaboration associated with care planning services. Information was gathered from patient records at the pharmacy, from patients’ electronic health records, and from other health care professionals such as nurses and physicians. At Sites 1 and 4, pharmacists accessed physicians’ medical care plans. Pharmacists at Site 1 routinely requested medical care plans directly from physicians’ offices. Some pharmacists at Site 4 were co-located with physicians at practice sites outside the community pharmacy. Pharmacists and physicians at Site 4 worked closely, sometimes meeting with patients together and co-developing care plan documents.

Methods of information sharing between pharmacists and physicians varied. The most frequent method involved pharmacists sharing a copy of the CACP with physicians after the care planning documentation was completed. To do this, they transmitted a facsimile of either the entire CACP document or a summary of the action items or issues outlined in the CACP. Transmission of information (faxing care plans to physicians) is required by the Compensation Plan. Physicians at Site 1 adjusted their office procedures to store the CACP within their information systems. Establishing processes for information sharing facilitated the building of relationships and supported care planning services. When pharmacists and physicians knew each other, the process worked more efficiently. For care planning services at the urban Site 2 and suburban Site 3, where pharmacists and physicians in the community did not have working relationships, frustration arose around information sharing. Other information sharing methods involved direct communication with physicians through telephone conversations, text messages, or face-to-face conversations.

The timing of information sharing was important. Most information sharing activities occurred after the care plan was completed. However, information sharing that occurred before completion of the care plan or through face-to-face communications was highly valued because clarifications could occur or more information gathered in those interactions. Some physicians valued information sharing at the time of development of care plans rather than receiving information after the fact. For example, a physician at Site 1 routinely contacted pharmacists to proactively share information and update documentation. Some physicians expressed frustration with pharmacist-developed care plans, citing instances of incomplete information, differences in opinions regarding drug therapy or prescribing decisions, or confusing documentation. Pharmacists and physicians that communicated directly about these frustrations worked together to improve information-sharing practices. Some adjustments resulted in tailored documentation to meet patient needs; for example, care plan summary templates were created to expedite the information sharing process. Care plans co-developed by pharmacists and physicians at Site 4 were valued by the physicians for their tendency to make them “think about things in a different way”.

Once patients were familiar with care planning services, they came to expect information sharing and collaboration from their pharmacists and physicians. Patients valued knowing that pharmacists and physicians shared information and communicated about their care. The value of care planning services was therefore associated with “interaction” and being “connected”.

#### 3.3.5. Revealing Possibilities for Pharmacists’ Contributions to Primary Health Care

*“I didn’t know that [the pharmacist] would talk to me about it. I thought I would just walk in, get my prescription and walk out, right? That’s the way I thought it was.”*

*(Site 4, Patient 1)*

Patients were generally unaware of the existence of care planning services prior to being approached by their pharmacist. Through interactions between patients and health care providers and increased familiarity with care plan documents, patients gained knowledge of community pharmacy services, for example, prescribing, that extended beyond the boundaries of traditional preparing and providing medications. Patients and physicians were long familiar with pharmacists’ dispensing services. However, expectations of the CACP were initially unclear. After gaining some experience with care planning services, one patient referred to the pharmacist as a “secret doctor” indicating that the abilities and expertise of the pharmacists were kept a secret until revealed through the CACP experience. Patients began to see pharmacists as primary health care providers who could do more than “fill prescriptions”; they began to appreciate the benefits they derived from the CACP, particularly receiving information about their medications and conditions. Providing care planning services revealed pharmacists’ drug therapy expertise and increased their interactions and information sharing with patients and other health care providers. Nurses and physicians viewed pharmacists as “an integral part” of primary health care teams. Physicians gained more respect for pharmacists’ roles and abilities. Despite their increased awareness of care planning services and pharmacists’ roles, patients did not refer to the CACP as a care plan, but rather as a “medication review”. The value of care planning services stemmed from having first-hand knowledge of and experience with pharmacists, revealing possibilities for their future contributions to primary health care.

#### 3.3.6. Meaning of Pharmacists’ Roles in Primary Health Care

*“I like what I do better, because it feels like I’m contributing more and I know I’m contributing more. And I appreciate being compensated for it. I’ve come to value it more appropriately as well … Now that we do have compensation for some of the clinical skills that we’re using all of the time, it makes it far more satisfying.” *

*(Site 2, Pharmacy Manager)*

As discussed earlier, pharmacists changed the focus of their work to provide care planning services, enabling them to co-create CACPs with patients and collaborate with other health care professionals. They spent time working with the CACP document template to gather information before meeting with the patient and to expedite the care planning and documentation processes. Increased interactions with people and their information fostered a sense of “doing more” for patients. As pharmacists gained experience with care planning services, they accepted more responsibility for patient care. While these activities (patient assessment, care planning, and documentation) were established as part of pharmaceutical care practice and gradually became embedded in their everyday work, there was a perceptible change in how they experienced their role. For some, the role felt “heavy” and “like I made a difference”. These changes were observed in association with the increased time pharmacists spent with patients, with the CACP document, and in activities related to information sharing. As well as feeling more connected to patients, pharmacists felt the CACP represented an investment because of the effort contributed to future encounters with patients; through involvement in the care planning process, pharmacists were positioned to support patients better through “rough times”. Pharmacists derived greater satisfaction with their work, felt that they were “contributing more” to patient care and primary health care, that they were staying “more current”, and that they enjoyed having “people depend on [them]”. These realizations, along with the material compensation associated with care planning services, changed how pharmacists experienced their role: with a renewed sense of responsibility, satisfaction, and meaning. 

## 4. Discussion

This study employed a novel theoretical approach to explore the perceived value of compensated care planning services provided by pharmacists. This approach placed the care plan document in the foreground. It also examined interactions between information and people. This study found that care plans provided material information, influenced everyday practice, linked people together, and mediated meaning-making. Care planning services were valued for their role in reinforcing patient-centered care, reducing waiting time for care, co-creating individualized plans with patients, collaborating with physicians and other health care providers, and revealing possibilities for pharmacists’ roles. This study also highlights pharmacists’ renewed sense of responsibility, satisfaction, and understanding of their role and potential contributions to primary health care. 

Through involvement with care planning services, everyday work by pharmacists and pharmacy staff changed in that more attention was focused on patients’ needs. Results echoed areas of value associated with primary health care services, namely accessibility, patient centredness, patient engagement, and experiences of health care providers [3,22,23,24,25]. These areas of value were also noted in prior research on care plans [19,54,55]. Similar to this current study, Council et al. [55] employed a prospective case study design to explore care planning by a group family practice at a community hospital in the US. In their setting, physicians, nurses, residents, and medical assistants all contributed to care planning. In that study, increased patient centredness, and patient involvement in the care plan were reported [55]. Thus, care plans bring value when patients are engaged in their development with primary care providers.

Through their participation in care planning services, patients valued having time and opportunity to talk with pharmacists about their medications and health concerns. Patients felt cared for and listened to. This experience represented a departure from patients’ past experiences. The difference was attributed to care planning. Brown et al. [54] summarized the essence of care planning as having a “better conversation”. The initial CACP interview became an ongoing conversation through various follow-up appointments at the pharmacy or telephone calls with patients; the conversation was often expanded to include physicians and others involved in the patient’s care. From patients’ perspectives, the conversation with pharmacists was less formal than speaking with physicians, like speaking with family members. This reflects the trust and confidence in the relationships built between patients and the pharmacists in this study. The results of this current study pointed to the unique conversation brought about by care planning services.

Previous research by Hindi et al. [19] found that care plans help to integrate community pharmacists into primary health care teams. Shared care plans, those co-developed by pharmacists and physicians, may further integrate community pharmacy in primary health care as well as address issues such as duplication of work and improve teamwork, which was seen in our study. Brown et al. [54] asserted the value of delivering a single coherent message to a patient in a shared care plan, describing how these plans support patients to engage in their own care and implement changes to their lifestyle. Experiences developing shared care plans in this current study were valued by patients, pharmacists, and physicians. Physicians valued the different perspectives and expertise brought by pharmacists as well as the efficiency of shared care plans. 

Information sharing is recognized as a significant part of care planning services [54]. In the current study, care plans facilitated coordination of information and patient care activities. Care plan documents, themselves, contribute patient information. With pharmacists contributing more and more to patient care in the primary health care system, care plan documents will become even more important for information sharing. However, lack of access to or incomplete patient information presents challenges for pharmacists providing care planning services. In Alberta, pharmacists have had access to provincial electronic health records, including laboratory tests and records of dispensed medications, since 2006 [28]. In the current study, complete information shared through documents or in conversation was highly valued and necessary to ensure accuracy and informed decision-making. Information systems, like NetCare in Alberta, are needed to support care planning services. Enhancements to the NetCare system that allow pharmacists to upload care plan documents to patients’ records will support easier information sharing between health care providers. Some pharmacists in this study lacked access to patients’ medical care plans. Having information from different perspectives of physicians and nurses would support pharmacists’ care planning services. In addition, information sharing mechanisms that support conversations between patients, physicians, pharmacists and other health care providers will bring further value to patients. Other research draws attention to the value of information sharing. Hindi et al. [19] conducted focus groups of patients, pharmacists, and general practitioners to identify how community pharmacy services may be better integrated within the primary care pathway for people with chronic conditions. Their results emphasized the need for robust information systems that included two-way flow of information. Council et al. [55] found that patient-centered care plans increased efficiency and the validity of information was enhanced as multiple team members and the patients were involved in construction of information. Methods to support information sharing contribute to collaboration and quality of primary health care services. Brown et al. [54] reviewed studies that evaluated care planning to develop a theoretically informed framework for how care planning works best. These investigators noted that sharing of written care plans within and across teams for patients with multimorbidity ensures consistency of messages. Attention to the quality of the care plan, along with completeness of information and consistency in presentation [19,56], may further enhance the value of information sharing associated with pharmacist care planning services and improve patient care.

Collaboration in the form of information sharing occurred in different ways at the sites included in this study depending on the individuals involved in care planning and the particular practice setting and location. Research by Talja [57] describes types and levels of collaborative information sharing related to documents. Talja’s 4 classifications relate to different goals and contexts and highlight the social aspects of information sharing including to: (1) provide information; (2) maximize efficiency; (3) form or strengthen relationships; and (4) develop novel approaches. In the context of the current study, information sharing by physicians and pharmacists at Site 4 produced shared care plans. This arrangement is an example of a novel information sharing strategy through which the relationships between pharmacists and physicians may be enhanced, and actions associated with care planning services in primary health care may be improved. Other strategies may also provide opportunities to expand information sharing strategies that support care planning services in primary health care. 

In the current study, care planning services were associated with gaining enhanced knowledge of community pharmacy services and identifying potential contributions to primary health care. Patients, physicians, and other health care providers initially had little knowledge of the nature and potential of care planning services. Moving forward, pharmacists’ involvement in care planning could add more value to the system if more individuals were aware of its intended aims and benefits. Latif et al. [58] reported that patients in the United Kingdom had poor awareness of the New Medicines Service provided by pharmacists. In their study, physicians were also unaware of the benefits of this service. Strategies are needed to educate patients about the value of the service to improve mutual understanding of policy among laypeople and professionals [58]. Expectations must be outlined for how the service is to be delivered, information shared, and anticipated outcomes. Providing patients with care plans may enhance patient experiences, improve knowledge of care planning services [59,60], and support goal setting, shared decision-making, and self-management [54].

The findings regarding pharmacists’ perceptions of the time required for care planning services were unexpected. Pharmacists in this study needed to spend significant time to develop a CACP, yet most regarded benefits of the care planning service as worthwhile for the patient and for themselves. In other research, the time and resources required to implement care planning services in community pharmacies represents a concern for pharmacists implementing similar services [61]. In contrast, pharmacists derived value from spending time to collaborate with patients and other health care professionals; involvement in care planning services sparked a renewed sense of responsibility, increased satisfaction, and gave meaning to their role. Despite the challenges associated with implementing care planning services, especially the time required for information sharing and documentation, pharmacists participating in this study reported high levels of satisfaction with their experiences. They also observed the value of CACPs in terms of compensation, interactions with patients, and engagement with others involved in the service. In addition, pharmacists perceived their role differently; they gained a greater sense of responsibility for patient care and their contributions to primary health care services. 

Given their access to information and prescribing authorization, the practice environment in Alberta supports changes in pharmacists’ roles and provides opportunities for them to contribute more to primary health care [35]. While pharmacists were well positioned to take on expanded roles in primary health care before the introduction of the Compensation Plan, care planning services were not prominent. Remuneration has been previously identified as a barrier to changing practice, implementing new services, and integrating pharmacists in primary health care [62,63,64,65]. Compensation directed towards valued patient care services may represent the final shift required for pharmacists to engage fully in patient care [66].

The strengths and limitations of this research must be considered when interpreting its results. Strengths of this research included the use of a longitudinal case study method that compared 4 sites, and combined multiple methods of data collection, materials, and perspectives. However, this study is limited in that it focuses on the practice environment in Alberta. Community pharmacy practice is diverse; thus, it is difficult to represent the entire range of experiences with care planning services. In Alberta, pharmacist involvement in care planning services has been fairly recently implemented and is still evolving. The number of care plans per month, which in this study was used as the variable representing experience with care plans, was self-reported and low at most sites. Finally, physician experiences and perspectives are integral to the question of value. Physician participation in the study was low; those that did respond may have had more favorable experiences with pharmacist involvement in care planning services. This selection bias should be addressed in future research confirming the results of this study.

In this study, an analytical focus on the social and material aspects of care planning services afforded an opportunity to explore the perceived value of compensated pharmacist care planning services, deepening our understanding of how care plans “made a difference” to patients, pharmacists, and other health care providers. The results of this study present implications for practice, policy, and research. Pharmacists may benefit from an understanding of how care plans make a difference to patients, the importance of the care plan document itself, possibilities for shared care plans, the importance of providing patients with copies of care plans, and how to align information sharing strategies with desired outcomes. Policy makers may consider providing compensation for care planning services that are most valued by stakeholders in order to derive the most benefit in primary health care. Including compensation for shared care plans and proactive and real-time information sharing, such as remuneration for phone calls, may be considered [19,55]. In the age of greater pharmacist engagement in primary health care, introducing compensation for new services may aid in timely and effective service delivery. Future research is needed to clarify the impact of compensation, level of reimbursement required to offset provision of care planning services, the processes and outcomes of shared care plans, how to provide patients with care plans, and various care plan documentation approaches [54,56].

## Figures and Tables

**Table 1:** Summary of patient eligibility criteria, information, and fees associated with Comprehensive Annual Care Plan (CACP) services [37,39].

	CACP ^1^	CACP Follow-up
Patient eligibility criteria	Two or more of the following chronic conditions: Hypertension Diabetes Chronic obstructive pulmonary disorder Asthma Heart failure Angina pectoris Ischemic heart disease Mental health disorder OR One of the above conditions plus 1 risk factor: Tobacco Obesity Addiction	CACP must have been completed. CACP Follow-up may be provided if a referral from a physician or hospital admission/discharge within 14 days of the CACP service OR A pharmacist determines that follow-up is needed.
Information gathered and recorded	Demographics Allergies and intolerances Health conditions Symptoms or signs to be treated Pregnancy or lactation status Medication use history/review (Best Possible Medication History—BPMH) Other health care products or devices Lifestyle factors—weight, tobacco use, illicit drug use, alcohol use, exercise Laboratory values Care plan—agreed goals of medication therapy, drug therapy problems, identification of possible interventions, plans for monitoring, and follow-up assessment	
Fees ^2^	$100 ^3^	$20 ^4^

^1^ CACPs—Comprehensive Annual Care Plans; ^2^ Canadian dollars; ^3^ Limit of 1 CACP per year; ^4^ Limit of 12 CACP Follow-up per year.

**Table 2:** Details of sites included in this study.

Description	Site 1	Site 2	Site 3	Site 4
Pharmacy type [53]	Independent	Franchise	Corporate	Independent
Population center [48]	Small ^1^	Large ^2^	Medium ^3^	Large ^2^
Pharmacists (with APA ^4^, injections authorization)	3 (3,3)	3 (1,3)	3 (1,3)	5 (4,5)
Registered technicians	1	-	1	2
Assistants	3	3	2	6
Pharmacy students	-	Periodically	Regularly	-
CACPs ^5^ completed per month	<20	<20	<20	>100

^1^ Population between 1000 and 29,999; ^2^ Population of 100,000 and over; ^3^ Population between 30,000 and 99,999; ^4^ APA—Additional Prescribing Authorization; ^5^ CACPs—Comprehensive Annual Care Plans.

**Table 3:** Interviews and observation hours.

Data	Site 1	Site 2	Site 3	Site 4	Total
Total number of interviews	27	15	16	19	77
Patient	11	5	8	5	29
Physician	2	2	-	2	6
Nurse	2	-	-	3	5
Pharmacy staff	4	4	2	3	13
Pharmacy student	-	-	2	-	2
Pharmacist ^1^	8	4	4	6	22
Hours of observation	24	28.5	26	15.5 ^2^	94

^1^ The number of pharmacist interviews exceeds the number of pharmacists participating in the study due to the fact that multiple interviews were conducted over the 12-month data collection period at each site. Some pharmacists were interviewed more than once; ^2^ The number of observation hours required at site 4 was less than other sites due to the higher number of care planning services observed.

## Appendix A

**Table A1:** Interview Topic Guide.

Participant	Topics
Patient	History with the pharmacy
	Experiences with care planning (CACP) services
	Perceptions and explanations of the value of care planning (CACP) services
	Added for site visit 3: Do you talk about your care plan goals? Do you have an action plan after a care plan has been developed for you?
Physician, Other Health Care Professional	History of the practice
	Experiences with the pharmacy
	Experiences with care planning (CACP) services provided by pharmacists
	How is the CACP stored?
	Perceptions and explanations of the value of care planning (CACP) services
Pharmacist, Pharmacy Technician, and Pharmacy Staff	History with the pharmacy
	Description of patient care services
	Experiences with care planning (CACP) services
	Implementation of care planning services
	Support provided/required to provide patient care services
	Benefits/challenges associated with provision of patient care services
	Changes, if any, to the professional role or activities of the pharmacy staff since the implementation of the Compensation Plan for Pharmacy Services
	Learning and professional development related to provision of patient care services
	Perceptions and explanations of the value of care planning (CACP) services
	Added for site visit 3: What information sources do you use for care planning (CACP) services? Do you have access to the medical care plan? What kind of feedback have you received on your care plans? How do you know if the care plan makes a difference? What professional development was helpful?

**Table A2:** Observation Form Topics.

Observational Data—Comprehensive Annual Care Plan (CACP) Services
**Observations**
Pre-CACP activities
Pharmacist–staff interactions
Patient–staff interactions
Patient–pharmacist interactions
Patient involvement in CACP development
Information seeking and use
Documents/forms/tools used
“Take away” documentation provided to the patient
Physician (or other) communication
CACP documentation sharing, storing
Post-patient interaction activities
Time spent on the activity
**Clarifications**
Questions to ask in interviews
**Description of the setting**
Location of staff in the setting, including changes over the course of the observation
Physical description of the setting
**Researcher reflexivity**
What changes to the observation process should be made for the next time?
How does the researcher feel about the day’s occurrences?
How do the observed actions compare to the findings from the document analysis? Do the key messages found there affect/influence/contradict the observations?
Did the researcher(s) have any apparent influence on the activities?

**Table A3:** Site-Specific Documents.

Site 1	Site 2	Site 3	Site 4
Care Plan Template (Example)	Care Plan Template	Care Plan Template	Care Plan Template
Initial Assessment with APA Template	Smoking Cessation Template	Smoking Assessment Template	Initial Assessment Template
Medication Sheet (Example)	Follow up Progress Notes (Example)	Adult Vaccination Assessment Template	Medication History Template
Alberta Health Services	Summary of Care Plan to a physician (Example)	Specific Disease-based Templates	Pharmacy Balance Care Plan Template
Care Plan Summary Template		Asthma Action Plan Template	Pharmacy Balance Patient Goal and Plan Template
Continuing Care Template			Letter to a physician (Example)
Alberta College of Pharmacy Documentation Templates			Pharmacist Prescribing Adaptation (Example)

**Table A4:** Value of Care Planning Services and Representative Quotes.

Value	Representative Quotes
Reinforcing patient-centered care	
	The value of it is a connection. The start of the connection. Before, you kind of got a connection with a patient sometimes if you listened to them over the counter and they told you their story … But now it seems like a given if you get the opportunity to sit down in the room with that patient. (Site 1, Pharmacy Manager)
	I feel like this… allowed [pharmacists] to prioritize what was important … I feel like I’ve been supported more. (Site 3, Pharmacy Manager)
	I like the structure and deliberateness. … [it] is a remarkable way to connect with people and to help them manage their health. It’s been really good that way to make that a deliberate process. (Site 2, Pharmacy Manager)
	This whole care plan process has made our pharmacists here do more for the patients. Like, we’re doing more—I’m not saying we weren’t doing the work before, but I feel like this, the whole care plan process has [compelled] us to really make sure that we’re following up with our patients with regards to a lot of medical conditions and medications that they start, you know, whereas we didn’t necessarily do that before. (Site 3, Pharmacy Manager)
	We build a relationship … That’s the foundation of my process of care…. So, instead of having my fulfillment come from any sort of outcome, it’s definitely attached to the process. (Site 4, Pharmacist 2)
	It’s [making] you close to patients—feel you’re close and patient. They phone and ask for [us] by names … they feel more comfortable talking to me or to [other pharmacists]. And you have more relationship with the patient. (Site 3, Pharmacist 1)
Reducing waiting time for care	
	I waited actually over 15 months to see a specialist with all my diabetes. The pharmacist in five minutes told me more than that specialist did, and there was no waiting period. (Site 3, Patient 1)
	You just felt more comfortable. With the doctor it’s more professional or, like I said, these [pharmacists] feel like family, you know. They’re easier to talk to. (Site 1, Patient 5)
	Sometimes you don’t have to go to the doctor’s appointment, you think, because [the pharmacist] helped you figure it out without doing that (Site 1, Patient 1)
	The doctor at the pain clinic was very busy all the time and you can’t get in to see him. You have to make an appointment a month or two ahead. And so it was easier for me to come and talk to the pharmacist who was talking back and forth with me at that time. [There was time] for him to sit down and tell me what exactly the situation [was]. (Site 4, Patient 1)
	There has been times when I have reacted to a medication, usually an antibiotic, on a weekend where I … was too sick to get to a doctor. And [the pharmacist] would advise me on what to do to get to the point where I could go to a doctor. (Site 2, Patient 5)
	[The pharmacist] takes the time to explain stuff to you. So I really appreciate that, because there’s not much of that anymore. (Site 4, Patient 5)
Co-creating individualized plans with patients	
	It made a difference to me. I mean, I appreciate it. I appreciate the chance, to go over my meds and things that bother me at the time or what we could do about one thing or another. (Site 1, Patient 4)
	When I call her regarding something, it’s very important. She always takes some time to listen. (Site 2, Patient 5)
	They listen to my concerns. Like, if I have any concerns about my medication somebody always takes the time to answer my questions. (Site 3, Patient 7)
	They’re not just your pharmacist, you know. They’re concerned about you too, you know. So that’s a good thing. … rather than just giving you pills like they used to. (Site 1, Patient 5)
	Well, my goals are to get my blood sugar down to an acceptable level because I’m Type 2 diabetes, and my blood sugar was way up in the 26 range. And with the help of [named pharmacist] and my family doctor, it is now down roughly about 6. (Site 3, Patient 7)
	Before, I abused myself. I didn’t care, right? And now, you know, this [success] is a result of just trying a few healthy things that [the pharmacist] has expressed interest that maybe I should think about doing. [It] has changed my life. Really. (Site 2, Patient 4)
Collaborating with physicians and other health care providers	
	Before, you had your doctor. Then this. Now it seems like they’re, you know, all connected. So it’s more interaction. Everybody isn’t out in the dark, you know. It seems like it’s better that way now. [Better] than it was before. (Site 1, Patient 5)
	It’s a wonderful adjunct to my practice. It makes my practice better… It makes me think about things in a different way. (Site 4, Physician 2)
	I know that with both [pharmacist and physician] they have my best interests at heart. They are working together to do the things that I need to have done. In fact, my doctor was saying the other day that the relationship I have with her, and she has with [the pharmacist], allows her to do and follow up on things that she normally wouldn’t have the chance to do. (Site 2, Patient 5)
	I talked to my doctor about my pharmacist and they said, “that’s good, we’ll talk back and forth”. It started out with the pharmacist talking with my doctor… back and forth, about my care. (Site 4, Patient 1)
	The value of someone’s follow-ups is unbelievable when you have different pharmacists rotating … I’ll even look back and it will be an interaction [with] the other pharmacist had in May … I’m dealing with the problem in September. I’ll understand how that went. (Site 1, Pharmacy Manager)
	The piece about talking to people and gathering information from them … to document that properly, really follow that up properly, and share the information properly so that other professionals I work with can be in on the story as well and can be part of that follow-up. (Site 2, Pharmacy Manager)
	The physician will outline a care plan for this patient… specific goals for therapy which I can then pull in to my care plans. I’m fortunate to be able to have access to those charts. (Site 4, Pharmacist 5)
	[The care plans] gets scanned into our EMR [electronic medical record] so that I can refer back to it at any point and also because we all share the same patients. Information is really important. (Site 1, Physician 2)
	We’re starting to get more of their care plans faxed to us. And because they’re doing [care plans, we are] getting more patients coming back to us with questions. And some of that’s very good because it seems we hadn’t realized he’d fallen by the way. And sometimes it’s a little bit of a nuisance because there’s a good reason why this patient isn’t on a statin and now we have to have this conversation all over again. (Site 2, Physician 1)
Revealing possibilities for pharmacists’ contributions to primary health care	
	I didn’t know that [the pharmacist] would talk to me about it. I thought I would just walk in, get my prescription and walk out, right? That’s the way I thought it was. (Site 4, Patient 1)
	Well, it was interesting. I’ve never done that [care plan] before, you know. The ones that give you the prescriptions are the doctors, and it seems like the pharmacy doesn’t know anything about it. But I found that I could get more information from [the pharmacist] than I could from a doctor. (Site 2, Patient 2)
	I don’t know if you are aware of this [pharmacist] but he’s a kind of a diabetes specialist … he gets my blood reports… he’s monitoring them …You’ve heard of a secret Santa? He’s kind of a secret doctor, you know. (Site 3, Patient 1)
	I think pharmacies are changing… I didn’t know that that [care planning] service was available, to tell you the truth. I always thought that, you know, pharmacists were just there to fill out the prescription, right? I didn’t think they really knew that much about what was going on. (Site 2, Patient 4)
	I have more respect for pharmacists now. Let’s be honest about it. I always thought they were just dispensing meds and whatever. (Site 4, Physician 2)
	I find that they are, have just become, an extension of my health care colleagues. (Site 4, Nurse Practitioner)
	This [care planning service] is a resource in limited situations like [this town]. It’s nice. We know we’re never going to have enough physicians to look after everybody properly like we should. So, it’s nice that the pharmacist can take some of that load. (Site 1, Physician 1)
Meaning of pharmacists’ roles in primary care	
	I like what I do better, because it feels like I’m contributing more and I know I’m contributing more. And I appreciate being compensated for it. I’ve come to value it more appropriately as well. … Now that we do have compensation for some of the clinical skills that we’re using all of the time, it makes it far more satisfying. (Site 2, Pharmacy Manager)
	I feel like even compared to a few years ago … I’ve noticed people depend on [me]—they come in more. They call you for more clinical questions. (Site 1, Pharmacist 2)
	It’s just like little light bulbs going off all throughout the day, it’s like I feel so good. I helped that person. I can’t believe what I just did. (Site 1, Pharmacy Manager)
	I’m more responsible. I feel more responsible for things than I ever did before. (Site 2, Pharmacy Manager)
	Being able to do at least a little bit of tracking [follow-up] to make me feel like I made a difference. (Site 4, Pharmacist 5)
	I honestly feel like the system [framework] that’s in place has allowed me to continue to stay more and more current because, you’re just looking at all the clinical stuff on a daily basis instead of just dispensing the medication… I think it definitely increased my knowledge and allowed me to stay a little bit more on top of the changes that occur. (Site 3, Pharmacy Manager)
	I have a responsibility to be good and to give [patients] quality and to continue to give good service. So, that kind of holds on you. It’s kind of heavy. (Site 4, Pharmacist 2)
